# The role of fear, closeness, and norms in shaping help towards war refugees

**DOI:** 10.1038/s41598-023-28249-0

**Published:** 2023-01-26

**Authors:** Małgorzata Kossowska, Paulina Szwed, Ewa Szumowska, Jolanta Perek-Białas, Aneta Czernatowicz-Kukuczka

**Affiliations:** 1 Institute of Psychology, Faculty of Philosophy, Jagiellonian University, Kraków, Poland; 2 Center for Evaluation and Public Policies Analysis, Institute of Sociology, Faculty of Philosophy, Jagiellonian University, Kraków Poland and Warsaw School of Economics, Kraków, Poland; 3 Institute of Religious Studies, Faculty of Philosophy, Jagiellonian University, Kraków, Poland

**Keywords:** Psychology, Human behaviour

## Abstract

The paper investigates the psychological factors associated with the unprecedented assistance that Poles have offered refugees from Ukraine since the outset of Russia’s invasion of Ukraine. Building on social identity theory, and examining the current social context in Poland, we focus on three social identity dimensions, i.e., a feeling of closeness towards refugees from Ukraine, anticipatory fears (of a Russian invasion), and a community norm of helping. These three dimensions predict collective helping resulting from a sense of a common fate and a feeling of togetherness with Ukrainians. We tested this hypothesis in a study (*N* = 1066) conducted between 11 and 17 March 2022. Participants were asked about their helping activities during the previous week; they also responded to questions on different measures of social identity processes. The results support our expectations, revealing that closeness, anticipatory fears, and social norms are associated with two forms of help: benevolent and activist. The results of the study contribute to the discussion on social identity processes underlying offers of help to people fleeing from war-zones. Thus, they enhance our understanding of the role of citizens in terms of their contribution to helping refugees, and can be used to improve responses to other humanitarian crises.

## Introduction

According to the United Nations High Commissioner for Refugees^1^, Russia’s invasion of Ukraine, beginning on the 24th of February 2022, has caused the world’s fastest-growing displacement crisis since World War II. In the first 6 weeks, over a quarter of Ukraine’s population fled their homes and an estimated 7.7 million people are now internally displaced. Most have decided to flee to Poland (3.5 million); significantly fewer of them to neighboring countries such as Romania, Hungary, Slovakia and Moldova. Almost overnight, Poland has gone from being one of Europe’s most homogeneous societies to hosting the world’s fourth-largest refugee population (after Turkey, Colombia, and the United States, respectively)^2^. As a result, population figures have swollen by 13% in the Polish capital Warsaw, 19% in Kraków, and 53% in Rzeszów^3^. A proportion of these Ukrainians have moved on to other European countries, but most have remained in Poland owing to cultural, linguistic, and/or family ties^4^. There is the desire among many of the refugees to be close to their country in the hopes that, when hostilities end, they can return^5,6^. Overall, the number of refugees flooding into Poland from Ukraine in the span of less than 5 weeks is more than double the number of those who, throughout 2015, fled wars, climate disasters and poverty, and reached Europe (Statistics in Immigration in EU^7,8^). Back then, receiving that unprecedented number of arrivals of refugees and migrants led to a crisis within the EU.

Currently, in the face of this influx, Polish officials are struggling to offer full assistance to all the people arriving in the country (see https://ec.europa.eu/migrant-integration/news/poland-parliament-adopts-law-assistance-ukrainian-refugees_en). By way of contrast, the humanitarian response from ordinary Polish citizens in this war emergency has been exceptional^3^. Indeed, it feels as if the entire country of Poland has joined the effort to welcome and comfort Ukrainian refugees^6,9^. Everyone from volunteer citizens and grassroots activists to local officials and civil society organizations have pulled together to provide refugees with a warm welcome. As a whole, they have played an important role in delivering support in every area of social life (such as housing, the labor market, social assistance, healthcare, language courses, education, training, legal aid, and counseling, as well as financial aid). This aid is proffered at border reception points and in other locations, mainly in large urban centers. Alternatively, on their own initiative, people have rushed to the border in private cars and buses to collect Ukrainian refugees and bring them to secure places^10^. On arrival in Poland, rooms in private homes are thrown open to those in need, with one study reporting that 17% of refugees from Ukraine were hosted in private homes^4,5^. As evidenced above, in the first days of the war, Poles offered two forms of help, i.e., ‘giving’ and ‘doing’ help^11^. The ‘giving’ form of support (i.e., benevolent) reflects efforts aimed at helping refugees through the transfer of money, goods, or services (synonymous with ‘charity’ or ‘philanthropy’). The ‘doing’ form (i.e., activist help) is social justice-oriented action; this form includes the provision of refugees with housing, collections of food/clothing/toys, or the organization of transport of refugees. It seems that in the current emergency situation in Poland, both benevolent and activist forms of help are equally offered to refugees from Ukraine^10^.

Although social support is common in emergencies^12,13^, in this particular context, it was completely unexpected, and the sense of disbelief was justified. First of all, before the Russian invasion of Ukraine, public opinion polls showed a rise in xenophobic attitudes towards Ukrainian migrants^14^. Secondly, during the Syrian refugee crisis of 2015, while under the government of the populist Law and Justice party (still in power as of 2022), Poland opposed plans to share asylum-seekers among the member states according to their country size^15^. Whereas Germany took in more than 1 M refugees from the Middle East, Poland refused the European Union’s request for it to accept a mere 7000^16^. Finally, in 2021, Poland deployed armed police and the military in order to force back a few thousand people from the border, mostly from Afghanistan and Syria, many of them women and children^17^. And according to opinion polls at the time, it was clear the public sided with the government’s stance^18^.

Given all this, how then is it possible that a country previously openly hostile in Europe to incoming refugees has all of a sudden become the most generous? Are helping behaviors toward refugees from Ukraine driven by sympathy for those who share the same ethnicity, culture, or religion? Or maybe, by helping Ukraine’s fleeing population, Poles are combating their own feelings of dread? What is the role of community norms? In our study, we aimed to answer these questions head-on, and thus contribute to improved responses to current and future humanitarian crises.

### State-of-the-art: social identity approach to helping

The social identity approach^19,20^ predicts that social psychological processes, contributing to a shared identity, predict the various forms of ‘helping others in need’ that can emerge. Shared identity causes people to act as members of a group, and to treat others as members of the same group, providing individuals with strengths and abilities that they lack as isolated individuals. Therefore, it may form the basis of a motivation to support cooperation, to assist others affected by an emergency, and also to bolster broader forms of solidarity such as courtesy^21^. In the current circumstances, the processes central to a shared identity are closeness (towards refugees), anticipatory fears of sharing a common fate (with the Ukrainians), and a social norm (that of helping); all factors trigger a special cognitive schema that causes people to accept more risk and less reward for helping others than they normally would^22–24^.

Indeed, the feeling of closeness towards others, defined in terms of a ‘small social distance’ to others, or ‘inclusion of the others in the self’^25,26^ has been shown to result in collective helping due to the perceived positive relationships between in- and outgroups, solidarity, or a feeling of togetherness, that all result in a strong collective resilience to danger^27–29^. Explaining the mechanism of this effect, researchers^30^ have put forward the following train of logic: when the ingroup member is part of the self, and the outgroup member is part of the ingroup member’s self, the outgroup member becomes part of the self. Thus, others are treated as a part of the ingroup and the self, i.e., as somebody who is close, and thus familiar. Importantly, in the face of threat, the stronger the feeling of closeness to others, the stronger the motivation to seek out the familiar rather than simply exit the situation, since the presence of familiar others has a calming effect, working against a “fight or flight” reaction^31^.

The feeling of closeness to others may be fueled by feeling anticipatory, (or prospective), fears related to a certain event^32^. Shared identity based on fear (e.g., of being invaded by Russia) increases the perception of a common fate, and this shared experience causes previous social group boundaries to dissolve^21^. Polish people indeed are reacting to Russia’s assault on Ukraine as if they themselves were at war^33^. Poland and Ukraine share a traumatic past and an anxious sense of themselves. It follows that the refugees from Ukraine might not just evoke memories of this intertwined past among Poles, but also warn them of an ominous shared future. The past is evoked because central and eastern European states have historically re-established their independence only to lose it a short time later^34^. The influx invokes a warning for the future because Poles are convinced that “sooner or later we will be next”, fueling a subcutaneous fear of the eventual return of dormant Russian imperialism^2^. It is worth noting that if it is indeed the case that a sense of a shared common fate is salient in a group in relation to an outgroup, then resolving that outgroup’s problem may become self-serving: by helping them, we are helping us. In light of evidence in the literature that prosocial behavior can be motivated by self-interest^23^, and the reasonable expectation that people are concerned with self-preservation, providing a framework that turns outgroup helping into self-serving acts should lead to a high endorsement of offers of assistance. The perception of an overarching common fate can transform former outgroup members into ingroup members, or at least people who share category membership at a superordinate level, and such shared category memberships have potent effects on behavior^35,36^.

In addition, this shared identity also leads to a situation where the behavior of relevant others can be a powerful (unintended) influence. When people with whom we share social identity express fear, or flee, or support others, this is likely to affect whether we do the same^21^. Indeed, the social identity approach suggests that humanitarian responses also take on a normative dimension when based on shared values or norms^37–39^. Some of these norms reflect pre-existing roles and rules that continue to operate, even during ‘extraordinary’ events^40,41^, whereas other norms are constructed within the emergency situation itself^42,43^. The social norms of helping could be thought of as moral norms, the critical difference being that they are not conditioned as strongly by social expectations as are social norms. Importantly, increasing the strength of norms can be induced by increasing people’s perceptions of the frequency of certain (e.g., solidarity-based) behaviors that close social network members are performing (descriptive norms) as well as what close social network members generally approve of (injunctive norms). Some research has even demonstrated that, where group norms are prosocial, people may actually compete to act more positively towards others^44^.

Moreover, in the context of helping refugees, some researchers have developed the subtle yet significant distinction between different forms of helping, i.e., ‘giving’ and ‘doing’. The ‘giving’ form of support, which could be labeled *benevolent*, reflects efforts aimed at helping through the transfer of money, goods, or services (and is synonymous with ‘charity’ or ‘philanthropy’) while the ‘doing’ form, i.e., *activist* support, is a form of social justice-oriented action. To distinguish the two, we can say that *benevolent* help compassionately alleviates the suffering of others, while *activist* help is driven by the desire to change current social, legal, and political structures to create greater equality^45^. Although in emergencies, both forms of helping are equally vital, we have reason to expect them to be underpinned by qualitatively different patterns of beliefs, attributions, and emotions with regards to humanitarian disadvantages^7,46^.

Summing up, although there is plenty of evidence that shared identity processes predict people’s helping behaviors, the phenomena have mostly been studied in the context of different kinds of disasters e.g., the sinking of the MV *Jupiter* (1988), the Hillsborough football stadium disaster (1989), the Accra [Ghana] football stadium crush (2001), and the Bradford football stadium fire (1985), or the devastation and aftermath of earthquakes and tsunamis^47,48^. Recently, however, a social identity perspective has been proposed to understand social conflicts^49^. This approach was applied in an ethnographic study exploring the experiences of psychosocial social support among a community of Syrian urban refugees in Jordan^50^. Therefore, since the role of shared identity processes in refugee emergencies is less well known, in this paper, we apply the social identity approach to this particular novel context. Our conjecture is that factors such as (a) closeness to others (here: to refugees), (b) fear of future events (here: an invasion of Poland by Russia), and (c) social norms of helping others in need, all contribute to a shared identity, and are the psychological bases of helping others in emergencies. We hypothesize that they allow groups in emergencies to both express and expect the values of solidarity and cohesion, and thereby coordinate and draw upon collective sources of support in order to deal with hardships and adversity^21^. In addition, we expect that feelings of closeness to people in need should be a core antecedent of *benevolent* support. On the other hand, *activist* support ought to be associated with intergroup processes such as the fear of a common fate, and social norms. We tested these hypotheses in a study conducted between 11 and 17 of March 2022, very soon after the start of the Russian invasion of Ukraine.

## Results

The study is part of a larger, longitudinal program, and we obtained additional results to the ones reported below. However, in this section, we report the results solely relevant to the theoretical claims of this paper.

To test our hypothesis, we performed logistic regression, with helping behavior (0—no help, 1—help) as a dependent variable, and psychological variables (i.e., closeness, anticipatory fear, and social norms) as predictors. We controlled for age, gender, education, financial situation, volunteer activity before the war, political ideology, and religiosity, as these variables were expected to be associated with helping behaviors. Vast swathes of research show that women, and well-educated and wealthier people, are more likely to help than will men, the less-educated or less well-off people in similar situations^51,52^. As far as political ideology and religiosity is concerned, given the negative stance of right-wing and religious Poles towards migrants from Ukraine prior to the war^17^, and their attitudes towards the plight of refugees from Belorussia^18^, we might expect that right-wing political ideology and higher religiosity would contribute to helping refugees less in the current emergency. Moreover, we might predict that any voluntary activity performed before the war could be closely associated with offering help to war refugees after the outbreak of the war.

As a first step, we focused only on the hypothesized psychological factors (i.e., closeness, anticipatory fear, and social norms, see Model 1 in Table 1). The results are in line with our expectations: participants who feel close to refugees from Ukraine (*b* = 0.02, Z = 1.02, *95% CI* [1.01;1.03]), fear that Poland will be attacked by Russia (*b* = 0.21, Z = 1.23, *95% CI* [1.07; 1.41]), and perceived that the social norm of ‘helping people in need’ exists in their community (*b* = 3.30, Z = 27.08, *95% CI* [16.81; 43.60] and *b* = 1.58, Z = 4.87, *95% CI* [3.13; 7.58], for strong and weak social norms of helping, respectively), were more prone to help (*Pseudo R2 [Negelkerke]* = 0.39).

**Table 1:** The results of the four models testing psychological predictors of helping.

		Model 1				Model 2				Model 3				Model 4			
		B	OR	Lower CI 95%	Upper CI 95%	B	OR	Lower CI 95%	Upper CI 95%	B	OR	Lower CI 95%	Upper CI 95%	B	OR	Lower CI 95%	Upper CI 95%
Intercept		− 4.38	0.12			− 2.66	0.70			− 6.22	.00			− 6.09	.00		
Social norm of helping	Strong norm of helping	3.30	27.08	16.81	43.60					3.04	20.90	12.79	34.15	3.03	12.79	12.79	33.72
	Weak norm of helping	1.58	4.87	3.13	7.58					1.42	4.14	2.63	6.53	1.42	2.63	2.63	6.46
	*Lack of norm (ref)*																
Anticipatory fear		0.21	1.23	1.07	1.41					0.26	1.13	1.11	1.51	0.26	1.30	1.12	1.49
Closeness towards refugees		0.02	1.02	1.01	1.03					0.02	1.02	1.01	1.03	0.02	1.02	1.01	1.03
Control variables																	
Age						0.01	1.01	1.00	1.02	.00	1.00	0.99	1.01				
Gender	Women					0.18	1.20	0.92	1.56	− 0.15	0.86	0.62	1.19				
	*Men (ref)*																
Education	Primary					− 0.41	0.67	0.46	0.96	− 0.31	0.73	0.48	1.12				
	Secondary					− 0.33	0.72	0.53	0.97	− 0.27	0.76	0.53	1.09				
	*Tertiary (ref)*																
Financial situation						0.26	1.30	1.16	1.46	0.20	1.22	1.06	1.41	0.24	1.27	1.11	1.45
Religiosity						0.33	1.39	1.23	1.56	0.20	1.22	1.06	1.40	0.16	1.17	1.06	1.30
Economic political beliefs						0.18	1.19	0.95	1.49	0.17	1.18	0.90	1.54				
Cultural political beliefs						− 0.25	0.78	0.65	0.93	− 0.02	0.99	0.78	1.24				
Being voluntary in the past						0.88	1.19	1.83	3.17	0.60	1.82	1.31	2.52	0.59	1.80	1.31	2.48
Pseudo R2 (Naegelkerke)		0.39				0.16				0.42				0.42			
N		1013				1042				1009				1009			

*OR* odds ratio.

Subsequently, we repeated the analysis by adding control variables to the model (see Model 3 in Table 1) and finally repeated the analysis with only these control variables that significantly predicted helping in Model 3 (see Model 4 in Table 1, *Pseudo R2 [Negelkerke]* = 0.42). It transpired that, although all of the control variables were significant (Model 2, Table 1), when added to the model with the main predictors (closeness, fear, and norm) such variables as age, gender, education, and cultural and economic-political beliefs became non-significant in predicting helping behaviors (see Models 3 and 4, Table 1).

We also hypothesized that closeness is associated with *benevolent* forms of helping, while anticipatory fear and social norms are linked to *activist* forms of helping. However, a chi-square test (*Chi*^*2*^ = 0.559, *P* = 0.110) revealed that participants devoted to one type of help were also most often engaged in the other one as well, with the distribution turning out to be similar (*H*(1) = 2.56, *P* = 0.110). The details of this analysis are presented in Table 2 and suggest that activist type of help coincided with benevolent type of help in our sample, at least in the initial stage of help mobilization, which we focused on in this study.

**Table 2:** Contingency table showing the relationship between benevolent (in columns) and activist (in rows) types of helping.

		Activists helping		Total
		No	Yes	
Benevolent helping	No	20	25	45
	% of activists helping	10.0%	6.3%	7.6%
	Yes	180	370	550
	% of activists helping	90%	93.7%	92.4%
Total		200	395	595
		33.6%	66.4%	100%

Thus, as the group of people delivering *activist* support was very small, but the groups of people delivering *benevolent,* and those offering *benevolent* & *activist* support at the same time were comparable, we decided to contrast these two groupings. By doing so, we could get deeper insight into the characteristics of the two groups. We thus ran a set of Mann–Whitney U tests (typically used to compare differences between two independent groups when the dependent variable is not normally distributed). These analyses showed that these groupings differ regarding age, gender, volunteering in the past, and in terms of the social norm of helping. Specifically, we found that people delivering both types of helping were younger than those delivering benevolent help only, they were more likely men (vs. women), with less experience with volunteering in the past, but with a stronger norm of helping in their network. These results are presented in Table 3.

**Table 3:** Differences between groups delivering different types of support.

	Activist and benevolent help (N = 370)	Benevolent help only (N = 180)	Value of U Mann–Whitney test (for independent samples) and p-value
Age	48.13 (16.20)	52.43 (16.03)	2.955 (0.003)
Gender [% of women]	38.3%	57.6%	− 4.231 (< 0.001)
Education [% of tertiary education]	43%	42.8%	− 0.374 (0.709)
Economical Political beliefs	2.48 (0.62)	2.52 (0.63)	− 0.784 (0.433)
Cultural political beliefs	2.68 (1.11)	2.70 (1.01)	− 0.276 (0.783)
Religiosity	4.47 (1.52)	4.59 (1.54)	− 1.103 (0.270)
Financial situation	3.78 (1.02)	3.68 (1.05)	1.116 (0.264)
Being voluntary in the past [% of helping in the past]	43.3%	53.4%	− 2.222 (0.026)
Social norms of helping [% of helpers the network]	8.7%	2.9%	− 2.003 (0.045)
Anticipatory fear	5.38 (0.98)	5.40 (1.02)	− 0.029 (0.977)
Closeness towards refugees	79.88 (13.67)	79.39 (14.14)	0.311 (0.756)

Values provided in columns are means with standard deviations (in parentheses) or percentages in a given group.

## Discussion

In this paper, we focused on the psychological dimensions underlying helping behaviors in the context of the refugee crisis in Poland. Our finding was that Poles’ helping behavior (in both forms, benevolent and activist) is predicted by their perceived closeness to the refugees from Ukraine, the prospective fear of being invaded by Russia, and social norms of helping people in need, with these factors holding after controlling for socio-demographic variables. Interestingly, however, such variables that are typically reported in the literature as strong predictors of helping such as age, gender, education, and cultural and economic-political beliefs lost their predictive power. In addition, the majority of our helping sample supported refugees in a *benevolent,* or *benevolent* & *activist* way, and these two groupings, although differing in terms of socio-demographics, were comparable when it came to social identity dimensions. This important finding shows that psychological rather than socio-demographic factors explain why people help others in need; thus, these factors should be taken into account when characterizing the type of people who respond to the need for refugee support, and when they do so.

In general, our findings are in line with the social identity approach suggesting that, in emergencies, processes contributing to shared social identity predict helping behavior. Thus, from this perspective, we may claim that the basis of widespread helping in this particular emergency is due to the emergence of a new social identity among people, arising from a feeling of closeness to the people in need, and perceptions of a common fate, in such a way that shared experience causes previous social group boundaries to dissolve. In addition, we may advocate that norms around the issue of helping others in the community contribute to a shared identity which becomes the basis for the provision of different types of social support, such as providing others with practical assistance (for instance, helping them to move furniture) and emotional support (for example, listening to others’ needs and comforting them). Indeed, it seems that shared identification allows groups experiencing emergencies to express and expect solidarity and cohesion, and to enhance mutual trust, and thereby increase their motivation to help.

The context of the study is important, as, before the war, Poles were highly reluctant to host refugees, even those fleeing from war^15^. Importantly, however, we showed that group processes contributed to a shared identity that might help to overcome the social barriers and release the energy and motivation needed to help those in straitened circumstances. This recognition tends to enhance the likelihood of the host residents responding with offers of help, and demonstrating helping behavior. Therefore, our results have some implications for policy and practice, stressing the need to understand, reinforce and work with (rather than against) social identity.

The study has some limitations. Firstly, based on previous findings, we assumed that the studied dimensions (i.e., closeness, prospective fears, and social norms) contribute to the identity shared with refugees from Ukraine. Although this assumption is fully justified, it was not tested empirically; any further study should highlight this issue. Secondly, the refugee emergency needs to be analyzed over the course of time. This is the case for any refugee crisis, and for this one in particular as well because the state of the war is in flux, and so in turn is the civilian situation of Poles and Ukrainians in Poland^4^. For example, the threat of being invaded by Russia among Poles, strongly felt at the outset of war, is probably much lower at the time of writing this (in June 2022, during a period of staunch resistance from Ukraine, supported by NATO and the EU). Thus, it would be sensible to assume that the feeling of common fate with Ukrainians has also decreased as well, as a result, as has the level of spontaneous help being offered. In addition, we may expect that Poland’s humanitarian mobilization may diminish under the influence of the anti-refugee sentiment of the current leadership, which has supported the extreme right-wing movement^2^. Next, some forms of helping behavior seem to be more beneficial for people than others, considering the timing. Our study was conducted at the very beginning of the war. Thus, Poles offered help directly following the refugees’ arrival in Poland, and were focused on satisfying basic human needs (namely: food, shelter, housing, clothes, medical care etc.). When these basic human needs are satisfied, for instance, some months after their arrival, the refugees might be more interested in receiving autonomy-oriented help and might better appreciate activism from the residents’ side^45^.

It is, however, possible that shared identity is constructed differently depending on the perception of the refugees. Some studies indeed demonstrated that the way people perceive refugees does affect their attitudes towards them^53^. If the participants have a different sense of refugee adversity (e.g., those fleeing war versus those in the country that they are fleeing to, but who are not directly affected by the conflict), it would affect how close they feel to them. Based on the survey by CBOS (72/2022) which tested Poles’ perceptions of refugees from Ukraine, we assumed that, at least at the time of the study, Poles perceived all refugees fleeing from the war as a homogenous group highly affected by the war. This issue still definitely calls for further study.

Furthermore, our study is correlational in nature (i.e., our predictor was not an experimental manipulation, thus the mediator did not clearly cause the outcome variable). Hence, we were unable to reliably test any causal relations^54^. However, in future studies, it will be important to test the role of shared identity as a mechanism underlying helping behavior.

As was mentioned, the mass displacement of people strongly influences the dynamics of entrenched communities, changing their ethnic proportions, affecting the economic situation as well as the culture and thinking of host communities. Given that an important factor in determining social dynamics is the specificity of relations constructed on the following axis: refugees—settled community—receiving country^3^, it was a limitation that there was no focus placed, in this study, on the role of different motives for giving help; nor was attention paid to the functions of the provision of help (e.g., preserving the refugees’ dependency or, conversely, facilitating their autonomy). In addition, there was no examination here of any implications of the unequal power relations between help providers and refugees^47^. Specifically, researchers make a distinction as to whether a helping behavior is autonomy-oriented or dependency-oriented; the former type of help enables help-seekers to solve the problem on their own having gained the required skills. In contrast, the latter type of help provides an immediate, full solution to a problem, and thus keeps help-recipients in a state of neediness. When considering the cultivation of a harmonious relationship between Poles and Ukrainians in Poland, a key element would be to know what kind of help is being offered. When people engage in dependency-oriented (as compared to autonomy-oriented) help toward refugees, they are more likely to be paternalistic and view the help-recipients as incompetent. In contrast, autonomy-oriented help toward refugees is more likely when individuals view refugees as competent. Researchers agree that autonomy-oriented help has more potential for social change than the dependency-oriented type^46^.

To conclude, although growing research suggests several psychological factors are at play in terms of producing helping behaviors toward refugees, we believe that in emergencies, social identity processes have real potential to enhance and strengthen different forms of helping behaviors, and hence afford displaced people’s safety, and promote their wellbeing. In our study, we showed that (a) the role of closeness towards refugees, (b) feelings of sharing a common fate induced by anticipatory fear in relation to the plight of refugees, and (c) norms of helping, predicted both forms of helping (i.e., both benevolent and activist) for the benefit of refugees from Ukraine. Thus, the results of the study not only contribute to the discussion on the psychological and contextual processes underlying helping people fleeing from war-torn regions, they also boost our understanding of the role of helping actions on the part of citizens, and this understanding can be used to improve better responses to other humanitarian crises.

## Method

### Participants

The study, constituting the first wave of a longitudinal project, was conducted between the 11 and 17 of March 2022, and participants were asked about their helping activities during the prior week. Study participants were recruited from a panel online population designed to represent the Polish population. The panel was guided by the research company Pollster Research Institute. In light of the topic of our research, the sample was balanced by gender, age, education, and place of residence in Poland (additional control was carried out for these regions which are closer to and more distant from the Polish–Ukrainian border). The minimum sample size was calculated in each region separately (treating regions as strata) as proportional to the population size of each stratum.

Among the panel members who met the inclusion criteria (*N* = 7697), a total of 1,081 (response rate: 14%) agreed to participate in this study (520 woman, 537 men, M_age_ = 47.29, SD = 0.52). The individuals who agreed to participate in the longitudinal panel study were included in this cross-sectional analysis but only fifteen participants were excluded from the analysis due to the too brief time they took to complete the survey compared to the average (about 6 min). As a result, the final sample included 1,066 participants (516 woman, 526 men, M_age_ = 47.50, SD = 0.52). Our sample was also well-balanced regarding education, i.e., 23.1% of participants declared primary education, 40.4%—secondary education, and 36.5%—higher education. In addition, 39.1% of participants declared that they had previously taken part in occasional volunteering prior to the war.

In our sample, 55.8% of participants declared their involvement in helping refugees from Ukraine during the first week of the war; 34.7% (*N* = 370) of participants who declared help, characterized it as benevolent and activist help, while 16.7% (*N* = 180) of them stated they had engaged solely in benevolent help, and 2.3% (*N* = 25) declared their engagement in solely activist help.

The sample size was determined before the data collection. We ran an a priori power analysis with the use of G*Power 3.1^55^. We assumed a medium effect size of *f* = 0.25, which revealed that a sample of 400 participants would be required to reach statistical power at the recommended 0.80 level^56^. Thus, our sample size was more than sufficient to run all the reported analyses. Importantly, however, as the study is a part of a larger project, we had to collect data from even more participants.

This program of study is approved by the Institute of Psychology Jagiellonian University Ethics Research Committee (KE/03/032020). The study procedure was in accordance with the ethical principles of the 1964 Declaration of Helsinki^57^. All participants gave informed consent to participate in the survey and could halt their participation at any point in time. All materials, scripts and data necessary for the replication of our results are available on the OSF page (for review purposes, the link is anonymized; https://osf.io/qzgdr/?view_only=ba444057fccd435dbf8abb1e8d2bddf5).

### Measures and procedure

For the sake of a larger research program, we administered several additional psychological measures (see materials on OSF); however, in this paper, we report only on those measures relevant to this paper.

#### Predictor variables

*Closeness towards refugees from Ukraine* was measured by the Social Distance Scale^25^. Participants were presented with five statements, each statement reflecting a smaller social distance than the previous one. Statements were assigned values from 5 (largest social distance) to 1 (smallest social distance). Sample items include: “I would agree that refugees should live in your locality” followed by “I would agree that refugees should live in your neighborhood”. Following the suggestions^57^ participants provided their answers on a 7-point Likert scale (1—definitely not; 7—yes, definitely). The answer provided for a given question was multiplied by the value of the question; next, the totals for each question were added to calculate the iScore index. The iScore can range from 15 to 105, with higher values indicating lower distance. The mean index in our sample was 75.43 (*SD* = 18.3).

*Prospective fear* was measured with a 7-item questionnaire, based on the Terrorism Catastrophizing Scale by^32^. All the items referred to the war in Ukraine or the threat posed by Russia (sample items: “I find it hard not to think about the threat posed by the war in Ukraine”; “I think the threat from Russia will never go away”; “I fear that the war in Ukraine will become more dangerous in the future”). Items were rated on a 7-point scale (from 1—strongly disagree to 7—strongly agree). Answers for the seven items were averaged to calculate a catastrophizing index (*M* = 5.23; *SD* = 1.14; Cronbach’s α = 0.87).

*Social norm of helping* We asked participants to determine how many people exist in their closest social network (family, friends, acquaintances), as well as how many people from their closest social network were currently helping refugees from Ukraine. We calculated the social norm of helping index by dividing the number of people helping by the total number of people in the social network, then multiplying the ratio by 100 to obtain percentages. We excluded participants who indicated a greater number of people in their network involved in helping than the total number of people in their network (n = 29), and participants who indicated unusually high numbers of people in their network (> 5025, n = 4). Thirteen participants did not provide any numbers. Thus, 1020 participants remained.

#### Dependent variables

##### Helping refugees from Ukraine

Firstly, we asked participants whether they had been involved in helping refugees from Ukraine during the previous week (answer: Yes or No). Those who answered Yes were presented with a 10-item questionnaire concerning different forms of help, divided into two subscales: benevolent help (5 items; sample item: “I financially support collections for refugees from Ukraine”), and activist help (5 items; sample item: “I transport refugees to places they need to go”). Answers within the subscales were averaged to calculate the indexes of benevolent help (*M* = 1.86; *SD* = 0.49; Cronbach’s α = 0.52) and activist help (*M* = 1.35; *SD* = 0.38; Cronbach’s α = 0.57).

#### Control variables

##### Religiosity

Participants filled in an 8-item questionnaire on religiosity^58^ that captures religious belief and devotion irrespective of religious denomination. Items were rated on a 7-point scale (from 1—strongly disagree to 7—strongly agree). All of the items are given here: “I have always believed in God”; “God judges my behavior and lifestyle”; ‘‘God created the universe,’’ ‘‘God could prevent suffering if He wanted to,’’ ‘‘I don’t believe death is the end,’’ ‘‘I find it hard to believe in God when there is so much [suffering]’’ (reverse- scored),‘‘The world would be a more peaceful place if people didn’t believe in God’’ (reverse-scored), and ‘‘Religion is a cloak for politics’’ (reverse-scored). These items formed an adequate index (*M* = 4.36; *SD* = 1.55; Cronbach’s α = 0.90).

##### Ideology

Participants’ political beliefs were assessed with the Political Beliefs Questionnaire^59^, which is comprised of cultural and economic beliefs subscales. The questionnaire consisted of 9 items for the cultural dimension, and 10 for the economic dimension, with 5-point answer scales (1 = strongly disagree, 5 = strongly agree). The higher the average score, the more right-wing the beliefs are in the domains of culture (*M* = 2.37; *SD* = 1.01; Cronbach’s α = 0.90), or the economy (*M* = 2.48; *SD* = 0.62; Cronbach’s α = 0.75). Both subscales were moderately negatively correlated (r = − 0.231, p < 0.001). It is worth noting that the negative correlations between cultural and economic beliefs are not unusual among Polish samples, or indeed in other contexts^60^.

*Perception of one’s financial situation* was measured by the question: “To what extent are you satisfied with your financial situation?” (1—extremely dissatisfied, 6—extremely satisfied; *M* = 3.51; *SD* = 1.15).

The participants completed an online survey, starting with the measures of helping refugees from Ukraine, then closeness, prospective fear, and social norms of helping. Following this came the questions on religiosity and political ideology. Finally, they were asked for their age, gender, level of education, level of volunteering before the war, and subjective financial situation, before being thanked and debriefed.

## Data Availability

The data that support the findings of this study are openly available in OSF at https://osf.io/qzgdr/?view_only=dcfbb331dc9b44a5966911c276609dec.
