# Publisher Correction: The role of fear, closeness, and norms in shaping help towards war refugees

**DOI:** 10.1038/s41598-023-31505-y

**Published:** 2023-03-21

**Authors:** Małgorzata Kossowska, Paulina Szwed, Ewa Szumowska, Jolanta Perek-Białas, Aneta Czernatowicz-Kukuczka

**Affiliations:** 1 Institute of Psychology, Faculty of Philosophy, Jagiellonian University, Kraków, Poland; 2 Center for Evaluation and Public Policies Analysis, Institute of Sociology, Faculty of Philosophy, Jagiellonian University, Kraków Poland and Warsaw School of Economics, Kraków, Poland; 3 Institute of Religious Studies, Faculty of Philosophy, Jagiellonian University, Kraków, Poland

Correction to: *Scientific Reports* 10.1038/s41598-023-28249-0, published online 26 January 2023

In the original version of this Article Jolanta Perek‑Białas was incorrectly affiliated with ‘Institute of Religious Studies, Faculty of Philosophy, Jagiellonian University, Kraków, Poland’. The correct affiliation is listed below.

Center for Evaluation and Public Policies Analysis, Institute of Sociology, Faculty of Philosophy, Jagiellonian University, Kraków Poland and Warsaw School of Economics, Kraków, Poland.

The original Article has been corrected.

